# Comparative analysis of aroma components and quality of *Geotrichum candidum* after space mutation breeding

**DOI:** 10.3389/fmicb.2022.908329

**Published:** 2022-07-22

**Authors:** Junjie Chen, Qianying Li, Jie Wang, Weizhe Chen, Qikai Zheng, Qingping Zhong, Xiang Fang, Zhenlin Liao

**Affiliations:** College of Food Science, South China Agricultural University, Guangzhou, China

**Keywords:** *Geotrichum candidum*, space breeding, HS-SPME-GC-MS, whole genome sequencing (WGS), relative odor activity value (ROAV)

## Abstract

**Aim:**

The aroma-producing strain of *Geotrichum candidum* GDMCC60675 was taken as the research object, the composition of aroma-producing substances of *G. candidum* was studied, and the target strains of *G. candidum* suitable for food additives were screened out by mutagenesis.

**Methods:**

Mutants were obtained by space breeding. The colony morphology and cell morphology of the mutant strain were identified, the phylogenetic tree of the two strains was constructed, and the whole-genome sequences of the wild strain and the mutant strain were compared. The aroma components and key odor compounds of the two strains were analyzed and compared by HS-SPME-GC-MS and E-nose detection, and the data were processed by using the relative odor activity value (ROAV) analysis method.

**Results:**

A mutant strain of *G. candidum* was found with different characteristics of aroma production compared with wild-type *G. candidum*. It was found that its colony morphology and cell morphology were similar. However, it was found that the aroma-producing substances produced by the two strains were different, and the key difference compound was phenyl ethyl alcohol, which also proved that the two strains were different, and the main aroma note was different.

## Introduction

Some aroma additives are added to food or cosmetics due to the sensory requirements of products. Many additives of flavors and fragrances are chemically synthesized or extracted from natural animals or plants (Yang, 2019), but these methods cannot guarantee their safety and sensory properties, and some raw materials in the process of synthesis and extraction are wasted; extractions of compounds from animals or plants are not widely used because of the high cost and low yield. Therefore, a method of microbial fermentation of aroma substances is new research and direction for the production of natural flavors and fragrances (Sun et al., 2008). Microorganisms have irreplaceable advantages such as fast growth and controllable growth conditions. There have been low requirements on microbial fermentation and extraction equipment, which can greatly save equipment costs. Therefore, flavors and fragrances produced by microbial fermentation have huge application prospects in the food or cosmetics industry. Among the microorganisms, *Geotrichum candidum* has gained scientists’ attention due to its ability to produce aroma substances.

*G. candidum* is a eukaryote with a morphology between yeast and mold (Sun, 2009), belonging to *Ascomycota*, *Saccharomycetes*, *Endomycetaceae*, and *Geotrichum* (Zhang, 2014). Its growth adaptability is strong, but its applications are relatively few, so the application of *G. candidum* fermentation in the production of flavors and fragrances has great application prospects.

Before this research, some studies have been conducted on the production of volatile substances by *G. candidum*. For example, *G. candidum* was first found to produce aroma substances during protein hydrolysis (Micheline and Lenoir, 1975); it was found to produce some fruity fatty acid methyl esters in 1982 (Koizumi et al., 1982; Sun et al., 2008); *G. candidum* ATCC 62217 also formed fruity aroma compounds (Daigle et al., 1999); and it was found that the aroma substances including ethyl acetate, propionic acid, and butyric acid also proved that the production of esters was related to organic acid metabolism and assimilation. In addition, the fermentation broth of *G. candidum* was analyzed by GC-MS, and the results were shown that the main fruity flavoring compounds of the *G. candidum* were esters and alcohols, and the relative content of ethyl acetate and benzyl alcohol was 9.5 and 1.6 g/L, respectively (Mdaini et al., 2006).

Environmental conditions such as microgravity and space radiation in space can provide excellent mutagens for microbial mutation breeding (Wang et al., 2014), and the perfect strains can be used in practical production. At present, the research on microbial space mutation breeding is mainly focused on the medical field (Changting, 2016; Middeleer et al., 2019), and there is little involved in the field of fermented food microorganisms. In the field of fermented food, aerospace microorganisms have been applied in baijiu, soy sauce, vinegar, and beer. For example, the fermentation of aromatic white wine was carried out by using the better hexanoic acid bacteria (45% increase in hexanoic acid yield) and aerospace cellar mud mixed with ordinary cellar mud in a certain ratio. The total acid content and total ester content of the resulting white wine were increased by 300 and 1,000 mg/L to 1,371 and 6,205 mg/L, respectively, and the most significant increase was in ethyl acetate, which was increased by 500 mg/L (Zhang and Zhuang, 2014). The use of aerospace mutagenized *Rhizopus* species *Aspergillus niger* and *Trichoderma reesei*, mixed with the originally used *Aspergillus oryzae*, resulted in the production of a raw soy sauce with good color, aroma, and taste, which met the requirements of high-quality raw soy sauce and increased the yield (Chen et al., 2011). The beers brewed with three strains of aerospace brewer’s yeast HT1, HT-2, and HT-3 showed higher contents of higher alcohols and esters than commercially available beers, and the content of diacetyl was controlled at about 0.08 mg/L (Yang et al., 2014). Therefore, aerospace mutagenesis breeding has great application prospects in the field of fermented food microorganisms.

Based on the aforementioned research status of flavors and fragrances, a strain of *G. candidum* GDMCC60675 has been isolated with special aroma characteristics. In this study, one or more *G. candidum* strains producing a pleasant aroma were purposefully screened and mutated by utilizing space microgravity mutagenesis breeding, and then the isolates are compared and analyzed by HS-SPME-GC-MS and E-nose combined with the method of relative odor activity value (ROAV). The purpose of that is to carry out basic research for the subsequent application of the strains in the food, health products, and cosmetics industries.

## Materials and methods

### Microorganism

Wild-type *G. candidum* GDMCC60675 was isolated from fermented soya beans, and it is stored in the authord soyaoratory.

### Culture media

Potato dextrose agar (PDB, 200 g/L of potato, 20 g/L of glucose, and 20 g/L of agar) and potato dextrose broth (PDA, 200 g/L of potato and 20 g/L of glucose) were used for the isolation, culture, and fermentation of wild-type and mutagenic-type *G. candidum*. (Ojokoh and Ekundayo, 2013).

Cells of wild-type *G. candidum* were stored on a strain preservation tube at −80°C and transferred to the PDA medium to refresh, before incubating at a 25°C incubator for 48 h, inoculating the mycelium or spores of *G. candidum* into 5 mL PDB medium at a 25°C incubator for 16–24 h, then inoculating that inoculum with 2% inoculation volume in 100 mL PDB medium to ferment, and putting it into a shaker incubator at 25°C for 16–24 h.

### Space breeding mutation

In this experiment, the target aroma-producing bacteria carried by China’s new spacecraft were used to observe the adaptability of the phenotype and genetic information of the strains to the extreme environment of space under the special microgravity (10^–6^–10^–3^ g), vacuum (101.325 kPa), temperature (17–23°C), and cosmic ionizing radiation (0.146 Gy/y) in space, to screen the strains with high-yield β-strains of phenyl ethanol or other volatile substances.

### Isolation and identification of the aroma-producing strains

#### Preliminary screening using sensory evaluation

The different strains of *G. candidum* were separated and purified by using the pure culture method. After space breeding mutation, sensory evaluation was performed in the mutagenic types *G. candidum* compared with wild-type *G. candidum* for primary screening, selecting one or several strains of *G. candidum* with special or obvious aroma-producing characteristics.

#### Morphological identification

Morphological characteristics of the wild-type and mutagenic-type *G. candidum* strains were examined by using an optical microscope and a scanning electron microscope.

Wild-type and mutagenic-type *G. candidum* strains were streaked in PDA plates, respectively, and cultured at 25°C for 48 h. Cell morphologies of each isolate were observed for the hyphae morphology, spore shape, and size using an optical microscope and a scanning electron microscope.

#### Molecular identification

##### Internal transcribed spacer sequencing

Total DNA extraction: for molecular analysis, the isolated colonies were grown for 2 days on PDA plates at 25°C. Crude template DNA was prepared as follows: An appropriate amount of mycelium was added to 10 μL extracting solution 1 [10 μL 5 mM Tris-HCL (pH 7.8), 1 mL 0.3 M NaOH]. The mycelium was broken using a pipette head and then heated at 95°C for 90 s. After that, extracting solution 2 [100 μL 5% (m/v) Triton-X100, 7.8 μL 0.3 M HCL] was added to an Eppendorf tube, then heated at 65°C during 30 min, and final froze at −20°C during 30 min. The supernatant fluids were used for polymerase chain reaction (PCR) amplification.

The rDNA-internal transcribed spacer (ITS) gene amplification: the PCR amplification of the rDNA-ITS gene was performed in a 25 μL reaction mixture containing 0.25 μL crude template DNA, 12.5 μL Taq PCR Master Mix (2×, with dye), 9.5 μL ddH_2_O 0.25 μL primer ITS1 (5′-TCCGTAGGTGAACCTGCGG-3′), and 0.25 μL primer ITS4 (5′-TCCTCCGCTTATTGATATGC-3′) purchased from Sangon Biotech (Shanghai, China) Co., Ltd. (Groenewald et al., 2012).

PCR Amplification Procedure: the reaction mixture was pre-denatured at 94°C for 2 min (denatured at 94°C for 1 min, annealed at 55°C for 1 min, extended at 72°C for 1 min) with 30 amplification cycles and finally kept at 72°C for 7 min, followed by cooling at 4°C.

The PCR samples were sent to Sangon Biotech (Shanghai, China) Co., Ltd. for sequencing after the DNA amplification was checked by electrophoresis. Then the ITS gene was blasted using the NCBI database.

##### Whole-genome sequencing

This study used a genomic approach to examine the molecular basis for aroma components of the two strains of *G. candidum*. Sending the whole genomes of the two strains to Tianjin Biochip Technology Co., Ltd. to sequence, single-molecule real-time (SMRT; Pacific Biosciences) and Illumina sequencing were used for *de novo* sequencing of the genome. Then the genomes of the two strains were sequenced, annotated, and compared to each other to determine the genomic features of volatile aroma compounds. Finally, finding the high-homology strains, the phylogenetic tree was constructed by using the Composition Vector Tree (CV Tree) in https://github.com/ghzuo/cvtree (Gz, 2021).

### Comparison and analysis of aroma components of two strains

#### Headspace solid-phase micro-extraction and gas chromatography-mass spectrometry analysis

The HS-SPME-GC-MS was used to extract and analyze the volatiles. The SPME fiber (PDMS-CAR-DVB, MA, United States) was pretreated in the GC injector at 250°C for 20 min at a 1.0 mL/min flow rate of carrier gas, and 5 g of samples were sealed in a 20-ml headspace vial and stirred at 400 r/min. After equilibrating in a 40°C water bath for 3 min, the aged SPME fiber was suspended 1.6 cm above the sample for 40 min and desorbed in the GC injector at 250°C for 5 min.

Compound analysis was performed using a Thermo Fisher Scientific VF-5ms GC (Thermo Fisher Scientific, Waltham, MA, United States) coupled with a Thermo Fisher Scientific Chromatography Data System (CDS) equipped with a DB-WAX capillary column (30 m × 0.25 mm × 0.25 μm, Thermo Fisher Scientific, Waltham, MA, United States). Helium (purity 99.999%) was used as the carrier gas at 1.0 mL/min. The oven was initially held at 40°C for 3 min, subsequently raised to 150°C at a rate of 4°C/min, and finally raised to 250°C at a rate of 8°C/min for 6 min. The electron ionization energy of the mass-selective detector was 70 eV. The chromatogram was recorded by monitoring the total ion currents in the m/z range of 50–450 atomic mass units (Moon et al., 2021).

The volatile compounds were identified with mass spectra in the NISTO5 Database. The data were then exported in an Excel format from the running software Thermo Scientific Xcalibur.

#### Electronic nose (PEN 3, Win Muster Airsense) analysis

A PEN3-type electronic nose was used, and the sensor array of the electronic nose composed of 10 metal oxide semiconductor field-effect transistor sensors, and different sensors had different sensitivities to different types of substances, as shown in Table 1 (Benedetti et al., 2008).

**TABLE 1:** Sensors and their applications in PEN3.

Number in array	Sensor name	General description
1	W1C	Aromatic compounds
2	W5S	Very sensitive, broad range sensitivity, react on nitrogen oxides
3	W3C	Ammonia, used as a sensor for aromatic compounds
4	W6S	Mainly hydride, selectively
5	W5C	Short-chain alkanes, aromatic compounds
6	W1S	Sensitive to a methyl group
7	W1W	Sensitive to many sulfides
8	W2S	Sensitive to alcohols, aldehydes, and ketones
9	W2W	Sensitive to aromatic compounds, organic sulfur compounds
10	W3S	Long-chain alkanes

The program was on the PC. A measurement cycle comprised an acquisition time of 120 s, delay time 60 s, and cleaning time 120 s, where the flow rate was kept at 1 mL/min. Then Win Muster software was used for principal component analysis (PCA), loadings analysis (LA), and linear discriminant analysis (LDA).

### Data analysis

#### Analysis of relative content of volatile aroma substances

The peak area corresponding to a complete dynamic headspace extraction was calculated to express the relative content of volatile aroma substances, and the calculation formula was as follows:

$$X\%=\frac{A_{i}}{\sum A_{i}}\times 100\%$$

where ∑*A*_*i*_ is the sum of all extractions from *A*_1_ to *A*, *A*_*i*_ represents the area of an aroma compound in a sample, and *X* represents each relative content of volatile aroma substances (Benedetti et al., 2008).

#### Analysis of relative odor activity value of volatile aroma substances

Due to differences in chemical composition, molecular structure, and degree of specific binding of olfactory receptor cells to compounds, people had different sensitivities to different compounds, and the lowest concentration at which a person can feel a substance is usually called the “detection threshold.” When the relative content was constant, the compound with a lower threshold was easier to be perceived; when the threshold was constant, the compound with a higher relative content was easier to be perceived. Therefore, the method combining the relative content of aromatic compounds with the detection threshold was used in this study to compare the relative odor contribution of each compound in the fermentation broth. So the ROAV was used to indicate the relative odor contribution of each compound to the overall fragrance of strains, and the calculation formula was as follows:

$${\text{ROAV}}_{\text{A}}\approx \frac{{\text{C}}_{\text{A}}}{{\text{C}}_{\text{stan}}}\times \frac{{\text{T}}_{\text{stan}}}{{\text{T}}_{\text{A}}}\times 100$$

where C_A_ and T_A_ are the relative content of flavor components and the corresponding detection threshold, respectively; C_stan_ and T_stan_ represent the relative content and corresponding detection threshold of the component that contributed the most to the overall fragrance of strains, respectively (Liu et al., 2008).

## Results

### Morphological identification of isolates

**FIGURE 1:**
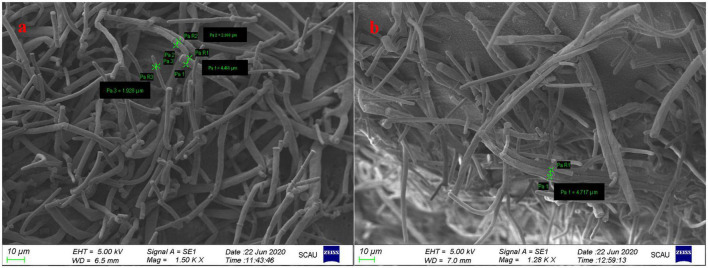
Scanning electron microscopy of two strains of *Geotrichum candidum*. Panel **(a)** shows wild-type *G. candidum* and **(b)** shows mutagenic-type *G. candidum.*

After preliminary screening and purification combined with sensory evaluation, one mutagenic *G. candidum* strain with an aroma-producing type different from that of the wild-type *G. candidum* was selected. Figure 1 shows scanning electron microscopy (SEM) of wild-type *G. candidum* and mutagenic-type *G. candidum*. According to the SEM image, most of the mutagenic-type *G. candidum* strains were wrinkled and thicker than those of wild-type *G. candidum*. No spores were observed because the spores attached to the mycelium were washed away when the cells were fixed.

**FIGURE 2:**
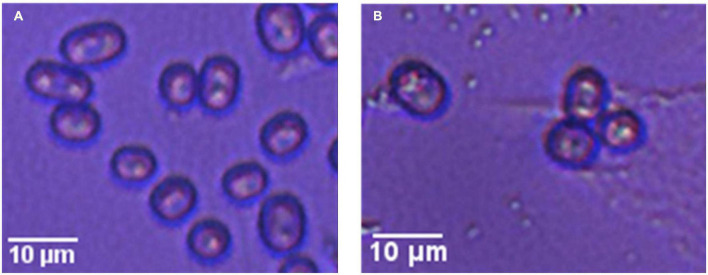
Microscopy images of two strains of *G. candidum*. Panel **(A)** shows the wild-type *G. candidum* and **(B)** shows mutagenic-type *G. candidum.*

Figure 2 shows microscopy images of the two strains; microscopically, it was observed that the spores of the two strains were oval in shape, but the spores of the wild-type strain were slightly larger than those of the mutagenic-type.

The morphological difference between the two strains may be due to changes in the growth and metabolism of the strain after mutagenesis, which also indicated that the strain had undergone mutagenesis.

### Molecular identification of isolates

#### Result of internal transcribed spacer sequencing

A 566-bp-size fragment of the rDNA-ITS gene was amplified and sequenced. NCBI database was used to compare the sequences, and then homology was compared. The sequence of the isolated strain had 100% similarity to the *G. candidum* strain (KY486784.1).

#### Result of the whole-genome sequencing

The wild-type *G. candidum* (24.09 Mbp; 1,54,410 reads, 15 contigs) had the similarity genomes to the mutagenic-type *G. candidum* (24.06 Mbp; 96,692 reads, 15 contigs). Accordingly, the wild-type (6,289 genes) and the mutagenic-type (6,272 genes) strains had a similar number of protein-coding genes. The two genomes had the same total GC content (41.44%). Second, we predict the difference in the biosynthesis pathway of secondary metabolites by counting the copy number of key enzyme genes, such as alcohols (phenyl ethyl alcohol) and esters. It was found that the copy numbers of the key enzyme genes of the Ehrlich pathway and shikimic acid pathway of the two strains were the same, which indicated that the copy numbers of Ehrlich pathway genes of the two *G. candidum* strains were the same at the gene level. However, there were two copies of malonic semialdehyde reductase (EC1.1.1.38) in the wild type and only one copy in the mutagenic type. Malate dehydrogenase was a key rate-limiting enzyme for the production of citric acid in the tricarboxylic acid cycle (TCA cycle), the reduction of its copy number in the mutagenic-type may slow down the TCA cycle, and the TCA cycle was a competitive approach of the Ehrlich pathway. The TCA cycle was a competitive pathway of the Ehrlich pathway (cinnamic acid pathway) and a final flow direction, so phenyl-ethyl alcohol could be detected in the mutagenic-type *G. candidum* because the expression of malate dehydrogenase was lower than that of wild-type *G. candidum*, which slowed down the TCA cycle; eventually, more L-phenylalanine flows to the Ehrlich pathway to produce phenyl-ethyl alcohol. In addition, mevalonate kinase (EC2.7.1.36) had 25 copies in the wild type, and only 24 copies in the mutagenic type. Mevalonate kinase was a key enzyme in the mevalonate pathway, which used acetyl-CoA as an initiator to synthesize isoprene pyrophosphate and dimethylallyl pyrophosphate. Acetyl-CoA was an important intermediate product in the metabolism of energy substances. Carbohydrates, fats, and proteins all flow into the TCA cycle through this.

**FIGURE 3:**
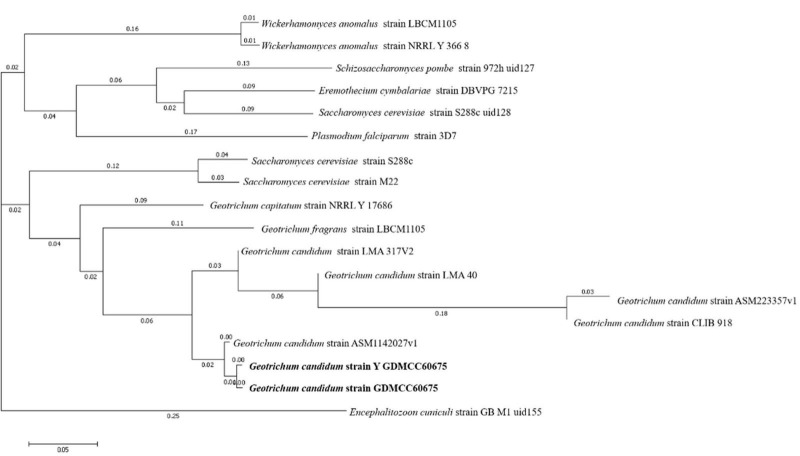
CV Tree of wild-type *G. candidum* and the mutagenic-type *G. candidum*.

Finally, to evaluate the phylogenetic relationships of the two strains’ genomes, a phylogenetic tree was constructed by using the method of composition vector tree (Figure 3). It was found that the two strains of *G. candidum* were most phylogenetically related to each other.

Combining molecular identification and morphological identification analysis of the isolate, it was proved that the strain used in the experiment was *G. candidum*. They had the closest genetic relationship with *G. candidum* strain ASM1142027v1. The NCBI Sequence Read Archive has accepted the raw whole genome of the sample with the number of PRJNA 823846.

#### Volatile aroma profiling of *G. candidum*

Table 2 details the relative content of volatile substances in the fermentation broth of two strains while PDA medium was used as the blank control group, according to HS-SPME-GC-MS analysis. Totally, 38 volatile substances were found in three groups.

**TABLE 2:** Relative content of volatile substances in three groups of samples.

Number	Volatile compounds	Odor description	Relative content/%		
			Blank control group	Wild-type	Mutagenic-type
1	Ethyl 3-methyl butyrate	Fruity	–	20.77	23.52
2	Ethyl hexanoate	Fruity	–	15.44	11.86
3	Ethyl isobutyrate	Fruity	–	8.03	8.48
4	Ethyl 2-methyl butyrate	Fruity	–	7.33	7.48
5	Methyl 3-methyl-2-butenoate	–	–	5.82	6.12
6	Ethyl 3-methyl-2-butenoate	–	–	5.20	4.43
7	Ethyl caprylate	Waxy	–	4.58	5.30
8	Ethyl caprate	Waxy	–	3.15	3.32
9	Butyl 3-methyl-2-butenoate	–	–	2.99	–
10	Butyl 3-methyl acetate	–	–	2.27	2.44
11	Ethyl heptanoate	Fruity	–	2.11	1.66
12	Isobutyl isovalerate	Fruity, green	–	2.10	–
13	Butyric acid ethyl ester	Fruity	–	1.88	2.00
14	Methyl isovalerate	Fruity	–	1.69	1.87
15	Methyl 2-methylbutyrate	Fruity	–	1.24	1.44
16	Ethyl valerate	Fruity	–	1.07	0.81
17	Isobutyl isobutyrate	Fruity	–	1.05	0.59
18	Butanoic acid, 2- methyl-, 2-methyl propyl ester	–	–	0.88	–
19	Methyl isobutyrate	–	–	0.80	–
20	2-hexenoic acid, ethylester	Fruity	–	0.76	0.66
21	Ethyl acetate	Ethereal	8.07	0.71	0.97
22	Ethyl *trans*-4-decenoate	Green, fatty	–	0.59	0.64
23	Isobutyl acetate	Fruity	–	0.34	0.35
24	Butyl 2-methyl acetate	–	–	0.31	0.36
25	Ethyl acrylate	Plastic	–	0.30	–
26	Ethyl nonanoate	Waxy	–	0.28	0.31
27	Ethyl (E)-2-octenoate	Fruity	–	0.27	0.31
28	Ethyl propionate	Fruity	–	–	0.81
29	Propanoic acid, 2- methyl-, 3-methylbutyl ester	Fruity	–	–	0.56
30	Phenethyl butyrate	Floral, fruity	–	–	0.30
31	1-pentanol	Fusel, fermented	17.86	1.29	1.51
32	2-methyl-1-butanol	Ethereal	6.68	0.92	1.06
33	Phenylethyl alcohol	Floral	–	–	7.63
34	2-ethyl-1-hexanol	Citrus, fatty	12.11	–	0.33
35	Hexyl alcohol	Herbal, green	8.23	–	–
36	Phenylacetaldehyde	Green, honey	3.92	8.98	0.46
37	Nonanal	Grassy, almond	37.60	–	–
38	Benzene	Fragrance, sweet	7.36	–	0.29

‘-’, mean no detection. The identification of volatile aroma components in this study was based on the SI/RSI index, and all SI/RSI were greater than 800. Odor description from Zhu et al. (2016); Liyan et al. (2020), Wang et al. (2020); YuanHui et al. (2020).

Only eight volatile substances were detected in the blank control group, mainly composed of alcohols (four species, a total relative content of 44.87%) and aldehydes (two species, a total relative content of 41.52%), of which nonanal had the highest relative content of 37.60%, but it was not detected in the other two groups, which proved that it was feasible to set the PDA medium as the blank control group.

Totally, 30 volatile substances were detected in the wild-type group, which mainly composed of 27 esters (a total relative content of 91.96%), two alcohols (a total relative content of 2.21%), and one aldehyde (a total relative content of 8.98%), of which the relative contents of five compounds were higher, including ethyl 3-methyl butyrate, ethyl hexanoate, phenylacetaldehyde, ethyl isobutyrate, and ethyl 2-methyl butyrate having the total relative content of 60.56%. Based on the aforementioned compounds, the total aroma types of the wild-type group are described as pineapple, apple, and other fruits.

In the mutagenic-type group, 31 volatile substances were detected, which mainly composed of 25 esters (a total relative content of 86.59%), four alcohols (a total relative content of 10.53%), and one aldehyde (total relative content of 0.46%), of which four of the top five compounds with a relatively high content were the same as the wild type, including ethyl 3-methyl butyrate, ethyl hexanoate, ethyl isobutyrate, phenyl ethyl alcohol, and ethyl 2-methyl butyrate accounting for 58.98% of the total volatile substances, while phenyl ethyl alcohol was not detected in the wild-type group. Based on the aforementioned composition of compounds, the total aroma types of the mutagenic-type group are described as rose and other florals.

From the aforementioned single-condition (relative content) analysis of the major contributors to aroma, it was known that esters and phenylacetaldehyde, and esters and phenylethyl alcohol were the major contributors to the aroma of wild-type *G. candidum* and mutagenic-type *G. candidum*, respectively. The two strains had almost similar volatile substances and relative contents, but phenylethyl alcohol was a unique compound in mutagenic-type *G. candidum*, having a rose-like floral. In addition, the relative content of esters in the wild-type group was higher than that in the mutagenic-type group.

#### Aroma quality relative odor activity value

First, the ROAV of the volatile substance that contributed the most to the total aroma of the sample to 100 was set, and then the ROAVs of each volatile substance were calculated. The volatile compounds that had an odor activity value (OAV) > 1 were commonly considered to be likely contributors to characteristic aromas, 0.1 < OAV < 1 was considered a modified odor compound, and OAV < 0.1 was considered a potential odor compound.

Saturated alkanes had a high detection threshold and were generally not likely to cause a significant odor sensation. Therefore, the relative odor contribution of esters, alcohols, and aldehydes to the overall aroma of the two strains was mainly analyzed. The ROAVs of the volatile substances are given in Tables 3, 4.

**TABLE 3:** Relative odor activity value (ROAVs) of volatile substances in the wild-type group.

Number	classification	Volatile compounds	Detection threshold (μg/kg)/(μg/L)	Relative content/%	References	ROAV
1	Esters	Ethyl 3-methyl butyrate	3 μg/L	20.77	a	8.62
2		Ethyl hexanoate	1 μg/L	15.44	b	19.23
3		Ethyl isobutyrate	0.1 μg/L	8.03	a	100
4		Ethyl 2-methylbutyrate	1 μg/L	7.33	c	9.13
5		Methyl 3-methyl-2-butenoate	3–5 μg/L	5.82	–	2.42–1.45
6		Ethyl 3-methyl-2-butenoate	1–3 μg/L	5.20	–	6.48–2.16
7		Ethyl caprylate	2 μg/L	4.58	a	2.85
8		Ethyl caprate	200 μg/L	3.15	d	0.02
9		Butyl 3-methyl-2-butenoate	2-2.2 μg/L	2.99	–	1.86–1.69
10		Butyl 3-methyl acetate	≈3 μg/L	2.27	–	≈0.94
11		Ethyl heptanoate	2.2 μg/L	2.11	b	1.19
12		Isobutyl isovalerate	≈2.2 μg/L	2.10		≈1.19
13		Butyric acid ethyl ester	1 μg/L	1.88	b	2.34
14		Methyl isovalerate	–	1.69	–	
15		Methyl 2-methylbutyrate	–	1.24	–	
16		Ethyl valerate	5 μg/L	1.07	b	0.27
17		Isobutyl isobutyrate	–	1.05	–	
18		Butanoic acid, 2- methyl-, 2-methylpropyl ester	–	0.88	–	
19		Methyl isobutyrate	7 μg/L	0.80	b	0.14
20		2-hexenoic acid, ethylester	–	0.76	–	
21		Ethyl acetate	5 μg/L	0.71	b	0.18
22		Ethyl *trans*-4-decenoate	–	0.59	–	
23		Isobutyl acetate	66 μg/L	0.34	b	0.06
24		Butyl 2-methyl acetate	–	0.31	–	
25		Ethyl acrylate	67 μg/L	0.30	b	0.01
27		Ethyl (E)-2-octenoate	–	0.27	–	
26		Ethyl nonanoate	–	0.28	–	
28		Ethyl Propionate	–	–	–	–
29		Propanoic acid, 2- methyl-, 3-methylbutyl ester	–	–	–	–
30		Phenethyl butyrate	–	–	–	–
31	Alcohols	1-pentanol	150.2 μg/kg	1.29	e	0
32		2-methyl-1-butanol	250 μg/kg	0.92	f	0
33		Phenylethyl alcohol	4 μg/kg	–	g	–
34		2-ethyl-1-hexanol	270000 μg/kg	–	b	–
35		Hexyl alcohol	500 μg/kg	–	f	–
36	Aldehydes	Phenylacetaldehyde	800 μg/kg	8.98	e	0.01
37		Nonanal	1 μg/kg	–	e	–

^a^From Zhao et al., 2014.

^b^From Leffingwell et al. (1991).

^c^From Tang et al. (2016).

^d^From Zhao (2016).

^e^From Gu et al. (2014).

^f^From Li et al. (2014).

^g^From Zhang et al. (2015).

‘-’mean no detection.

**TABLE 4:** Relative odor activity values of volatile substances in the mutagenic-type group.

Number	Kind	Volatile compounds	Detection threshold (μg/kg)/(μg/L)	Relative content/%	References	ROAV
1	Esters	Ethyl 3-methyl butyrate	3 μg/L	23.52	a	9.25
2		Ethyl hexanoate	1 μg/L	11.86	b	13.95
3		Ethyl isobutyrate	0.1 μg/L	8.48	a	100
4		Ethyl 2-methylbutyrate	1 μg/L	7.48	c	8.82
5		Methyl 3-methyl-2-butenoate	3–5 μg/L	6.12	–	2.41–1.44
6		Ethyl 3-methyl-2-butenoate	1–3 μg/L	4.43	–	5.22–1.74
7		Ethyl caprylate	2 μg/L	5.30	a	3.13
8		Ethyl caprate	200 μg/L	3.32	d	0.02
9		Butyl 3-methyl-2-butenoate	2–2.2 μg/L	–	–	–
10		Butyl 3-methyl acetate	≈3 μg/L	2.44	–	≈0.96
11		Ethyl heptanoate	2.2 μg/L	1.66	b	0.89
12		Isobutyl isovalerate	≈2.2 μg/L	–		–
13		Butyric acid ethyl ester	1 μg/L	2.00	b	2.36
14		Methyl isovalerate	–	1.87	–	
15		Methyl 2-methylbutyrate	–	1.44	–	
16		Ethyl valerate	5 μg/L	0.81	b	0.19
17		Isobutyl isobutyrate	–	0.59	–	
18		Butanoic acid, 2- methyl-, 2-methylpropyl ester	–	–	–	–
19		Methyl isobutyrate	7 μg/L	–	b	–
20		2-hexenoic acid, ethylester	–	0.66	–	
21		Ethyl acetate	5 μg/L	0.97	b	0.23
22		Ethyl *trans*-4-decenoate	–	0.64	–	
23		Isobutyl acetate	66 μg/L	0.35	b	0.01
24		Butyl 2-methyl acetate	–	0.36	–	
25		Ethyl acrylate	67 μg/L	–	b	–
26		Ethyl nonanoate	–	0.31	–	
27		Ethyl (E)-2-octenoate	–	0.31	–	
28		Ethyl propionate	–	0.81	–	
29		Propanoic acid, 2- methyl-, 3-methylbutyl ester	–	0.56	–	
30		Phenethyl butyrate	–	0.30	–	
31	Alcohols	1-pentanol	150.2 μg/kg	1.51	e	0
32		2-methyl-1-butanol	250 μg/kg	1.06	f	0.01
33		Phenylethyl alcohol	4 μg/kg	7.63	g	2.25
34		2-ethyl-1-hexanol	2,70,000 μg/kg	0.33	b	0
35		Hexyl alcohol	500 μg/kg	–	f	–
36	Aldehydes	Phenylacetaldehyde	800 μg/kg	0.46	e	0
37		Nonanal	1 μg/kg	–	e	–

^a^From Zhao et al., 2014.

^b^From Leffingwell et al. (1991).

^c^From Tang et al., 2016.

^d^From Zhao (2016).

^e^From Gu et al. (2014).

^f^From Li et al. (2014).

^g^From Zhang et al. (2015).

‘-’mean no detection.

The ester compound was an aromatic compound produced in many fermentation processes, and its detection threshold was very low, so it can provide a characteristic aroma even when the relative content was very low. Because short-chain esters have a strong polarity and are easily dissolved in water and ethanol, and the detection threshold was higher. Short-chain esters have a characteristic that as the carbon chain lengthens, water solubility and detection threshold decrease, and hydrophobicity increases. Then, as the carbon chain grows, the boiling point and detection threshold increase, volatility decreases, and the carbon chain increases. In addition, when the carbon number was the same, branched chain esters have a smaller detection threshold than linear chain esters (Fang and Xu, 2011). The analysis showed that branched medium- and long-chain esters had a smaller detection threshold. So, in this study, considering the relative content of esters or its detection threshold, namely, ROAV, the esters having a higher relative content were the key odor compounds of these two strains.

Among alcohol compounds, it was generally analyzed the relative odor contribution of unsaturated alcohol because it had a lower detection threshold than other alcohols. In this study, rose-like floral phenethyl alcohol, which was fermented by mutagenic-type *G. candidum*, reached a relative content of 7.63% and had a lower detection threshold. Many aldehydes also had lower detection thresholds. For example, hyacinth-like floral phenylacetaldehyde produced by mutagenic-type *G. candidum*, so it was not the key odor compound of the mutagenic type.

Before analyzing the ROAVs of volatile substances, the unknown detection threshold of other esters with a high relative content can be inferred according to the relationship between the ester carbon chain length and the detection threshold. For example, the detection threshold of methyl 3-methyl-2-butenoate (C_6_H_10_O_2_) was between that of ethyl 3-methyl butyrate (C_7_H_14_O_2_) and ethyl acetate (C_4_H_8_O_2_), which was 3–5 μg/L; the detection threshold of ethyl 3-methyl-2-butenoate (C_7_H_12_O_2_) was between that of ethyl 3-methyl butyrate (C_7_H_14_O_2_) and ethyl hexanoate (C_8_H_16_O_2_), which was 1–3 μg/L; the detection threshold of butyl 3-methyl-2-butenoate (C_9_H_16_O_2_) was between that of ethyl caprylate (C_10_H_20_O_2_) and ethyl heptanoate (C_9_H_18_O_2_), which was 2–2.2 μg/L; the detection threshold of butyl 3-methyl acetate (C_7_H_14_O_2_) was approximately equal to that of ethyl 3-methyl butyrate (C_7_H_14_O_2_), which was 3 μg/L; the detection threshold of isobutyl isovalerate (C_9_H_18_O_2_) was approximately equal to that of ethyl heptanoate (C_9_H_18_O_2_), which was 2.2 μg/L.

Tables 3, 5 show the ROAVs of 30 important volatile compounds in wild-type *G. candidum*. It was observed that 11 volatile compounds were key odor compounds, and the relative odor contribution ranking is given in Table 5: ethyl iso-butyrate > ethyl hexanoate > ethyl 2-methyl butyrate > ethyl 3-methyl butyrate > ethyl 3-methyl-2-butenoate > ethyl caprylate > methyl 3-methyl-2-butenoate > butyric acid ethyl ester > butyl 3-methyl-2-butenoate > ethyl heptanoate > isobutyl isovalerate, all of which are esters. The ROAVs of two alcohols and one aldehyde detected were very small or insufficient to count, so alcohols and aldehydes were not the key odor compounds in the wild-type. In addition, there were eight modified odor compounds and 11 potential odor compounds in wild-type *G. candidum*.

**TABLE 5:** Analysis of key flavor compounds in the two strains.

	Wild-type	ROAV	Mutagenic-type	ROAV
Key odor compounds	Ethyl isobutyrate	100	Ethyl isobutyrate	100
	Ethyl hexanoate	19.23	Ethyl hexanoate	13.95
	Ethyl 2-methyl butyrate	9.13	Ethyl 3-methyl butyrate	9.25
	Ethyl 3-methyl butyrate	8.62	Ethyl 2-methyl butyrate	8.82
	Ethyl 3-methyl-2-butenoate	6.48	Ethyl 3-methyl-2-butenoate	5.22
	Ethyl caprylate	2.85	Ethyl caprylate	3.13
	Methyl 3-methyl-2-butenoate	2.42	Methyl 3-methyl-2-butenoate	2.41
	Butyric acid ethyl ester	2.34	Butyric acid ethyl ester	2.36
	Butyl 3-methyl-2-butenoate	1.86	Phenylethyl alcohol	2.25
	Ethyl heptanoate	1.19		
	Isobutyl isovalerate	1.19		
Modified odor compounds	Butyl 3-methyl acetate	0.94	Butyl 3-methyl acetate	0.96
	Ethyl valerate	0.27	Ethyl heptanoate	0.89
	Ethyl acetate	0.18	Ethyl acetate	0.23
	Methyl isobutyrate	0.14	Ethyl valerate	0.19
	Isobutyl acetate	0.06	Ethyl caprate	0.02
	Ethyl caprate	0.02	Isobutyl acetate	0.01
	Ethyl acrylate	0.01	2-methyl-1-butanol	0.01
	Phenylacetaldehyde	0.01		
Potential odor compounds	1-pentanol	<0.01	1-pentanol	<0.01
	2-methyl-1-butanol	<0.01	2-Ethyl-1-hexanol	<0.01
	Methyl isovalerate	–	Phenylacetaldehyde	<0.01
	Methyl 2-methylbutyrate	–	Methyl isovalerate	–
	Isobutyl isobutyrate	–	Methyl 2-methylbutyrate	–
	Butanoic acid, 2- methyl-, 2-methyl propyl ester	–	Isobutyl isobutyrate	–
	2-hexenoic acid, ethylester	–	2-hexenoic acid, ethylester	–
	Ethyl *trans*-4-decenoate	–	Ethyl *trans*-4-decenoate	–
	Butyl 2-methyl acetate	–	Butyl 2-methyl acetate	–
	Ethyl nonanoate	–	Ethyl nonanoate	–
	Ethyl (E)-2-octenoate	–	Ethyl (E)-2-octenoate	–
			Ethyl Propionate	–
			methyl-, 3-methylbutyl ester	–
			Phenethyl butyrate	–

‘-’ mean no detection.

Similarly, Tables 4, 5 show that there were 31 volatile compounds detected in the mutagenic-type *G. candidum*. It was analyzed that the ROAVs of eight esters and one alcohol were greater than 1, so there were nine key odor compounds among them in the mutagenic-type, and the relative odor contribution ranking is shown in Table 5: ethyl iso-butyrate > ethyl hexanoate > ethyl 3-methyl butyrate > ethyl 2-methyl butyrate > ethyl 3-methyl-2-butenoate > ethyl caprylate > methyl 3-methyl-2-butenoate > butyric acid ethyl ester > phenylethyl alcohol. In addition, there were seven modified odor compounds and 14 potential odor compounds in mutagenic-type *G. candidum*.

From the aforementioned method combining the relative content and the detection threshold of volatile compounds in two strains, it was analyzed that the types of volatile compounds detected by the two strains were similar, and esters were the main aroma-producing substances among them, such as ethyl iso-butyrate, ethyl hexanoate, ethyl 3-methyl butyrate, ethyl 2-methyl butyrate, ethyl 3-methyl-2-butenoate, ethyl caprylate, methyl 3-methyl-2-butenoate, butyric acid ethyl ester, ethyl heptanoate, and isobutyl isovalerate. The two strains had similar relative odor contributions to the aforementioned substances, so it can be concluded that the two strains were similar in the main types of aromas, both of which were based on floral and fruity aromas.

However, it was found that butyl 3-methyl-2-butenoate, isobutyl isovalerate, and phenylethyl alcohol were only detected in the two strains, respectively, therefore, it is speculated that these three volatile compounds were the key compounds that cause different aromas of the two strains: butyl 3-methyl-2-butenoate and isobutyl isovalerate produced by the wild-type *G. candidum* with fruity aromas, and phenylethyl alcohol produced by the mutagenic-type *G. candidum* with rose-like floral. The total aroma notes of the two strains showed fruity and floral-like aromas, respectively. The estimation of phenylacetaldehyde based on the relation between the relative content and relative odor contribution in 2.3.1 was a key odor compound that was wrong.

#### Result analysis of the electronic nose

##### Principal component analysis

**FIGURE 4:**
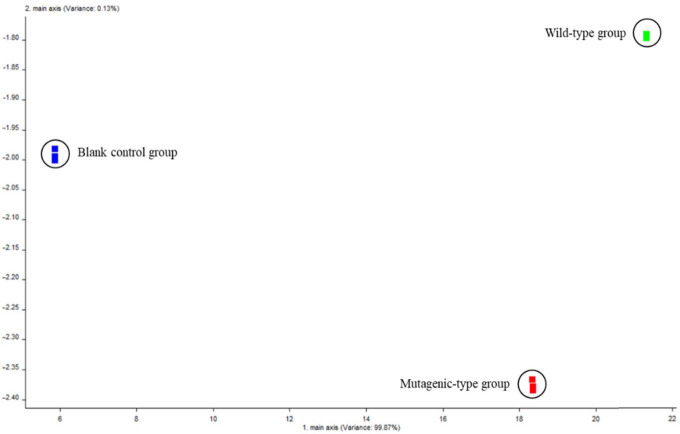
Principal component analysis chart of volatile components of three groups of samples.

To highlight the relative odor contribution of each volatile substance generated by the two isolated strains, a PCA was performed (Figure 4). Figure 4 shows that analysis of the highest eigenvalues for the first two principal components (PC1 and PC2) allowed us to account for 100% of the total variance. PC1, which accounted for 99.87% of the total variance; and PC2, which accounted for 0.13%, indicated that the two principal components had represented the main information characteristics of the sample. The data indicated that one blank control group, the wild-type group, and the mutagenized-type group were far apart, which showed that there was a large difference in volatile components between the three groups.

In addition, comparing the wild-type group and the mutagenized-type group with the blank control group, it was found that the relative position distance of the PC1 was significantly large, while the relative position distance of the two strain groups was obviously large on PC2. It again proved that it was feasible to use PDA as the blank control group.

##### Linear discriminant analysis

**FIGURE 5:**
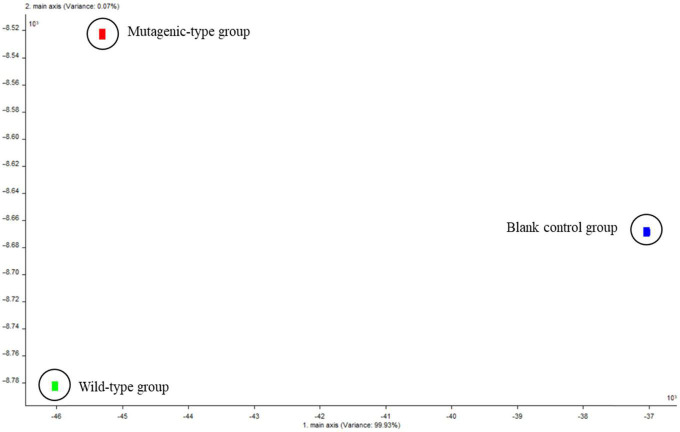
Linear discriminant analysis chart of volatile components of three groups of samples.

The LDA of the three groups from Figure 5 can obtain similar results as PCA, and the total variance (LD1 and LD2) accounted for 100%. LD1, which accounted for 99.93% of the total variance; LD2, which accounted for 0.07%; and the blank control group and the two groups of samples were far apart, which showed that there was a large difference in volatile components between the three groups. Summarizing the PCA and LDA, it again proved that the composition of volatile substances produced by the two strain groups was different.

##### Analysis of sensitivity to sensors with radar

**FIGURE 6:**
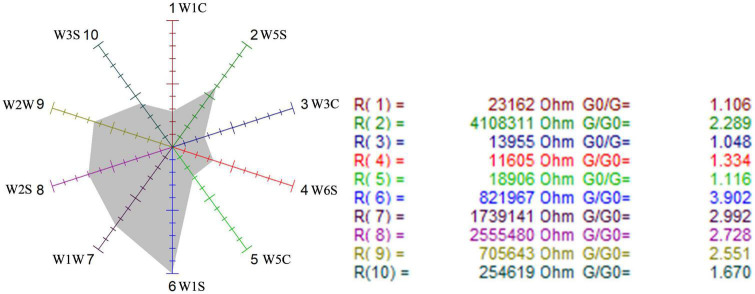
Odor sensor radar of the blank control group.

**FIGURE 7:**
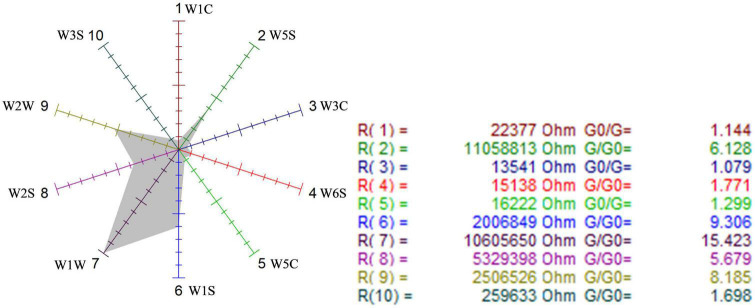
Odor sensor radar of wild-type *G. candidum*.

**FIGURE 8:**
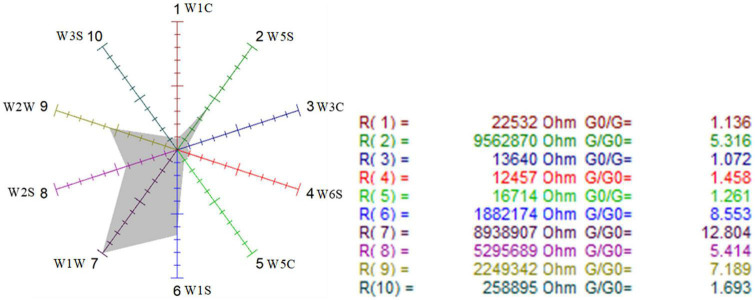
Odor sensor radar of mutagenic-type *G. candidum*.

First, the sensor radars were used to visually analyze the sensitivity of the three samples to the 10 sensors. Figures 6–8 show that the odor sensor radar of the blank control group, wild-type group, and mutagenized-type group.

The sensors with strong responses in the blank control group were W1S, W1W, W2S, and W2W, that is, methyl group, sulfides, alcohols, and aromatic substances. In the wild-type group, sensors W1W, W1S, W2W, W2S, and W5S respond strongly, that is, the wild-type mainly contained sulfides, methyl group compounds, aromatic compounds, alcohols, aldehyde ketones, and nitrogen oxides. W1W, W1S, W2W, W2S, and W5S sensors also responded strongly in the mutagenic-type group, but the radar area was larger than that in the wild-type, which indicated that the mutagenic-type mainly contained the same compounds as the wild-type, but the content increased. The same result was also concluded by HS-SPME-GC-MS, which analyzed that the mutagenic-type strain detected more alcohol than the wild-type strain.

Compared with the blank control group and the two strains, the response value of the sensor W1W (sensitive to sulfide) in the wild-type and the mutagenic-type was 5.15 and 4.27 times higher than that in the blank control group, separately, indicating that after fermentation with *G. candidum*, sulfide increased significantly.

##### Analysis of sensitivity to sensors with loadings

The loadings analysis method was used to analyze the sensitivity of the three samples to the 10 sensors, to distinguish the contribution rate of the 10 sensors of E-nose to the samples and the relative importance of each sensor.

**FIGURE 9:**
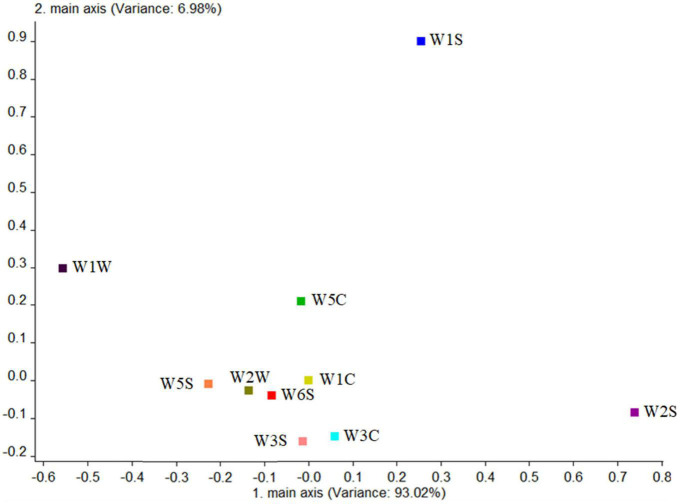
Loadings analysis chart of the odor sensors of the blank control group.

First, the blank control group was analyzed. Figure 9 shows that the total variance accounted for 93.02%. The W2S sensor (sensitive to alcohols, aldehydes, and ketones) had the largest contribution rate in the first principal component, while in the second principal component, the largest contribution rate was the W1S sensor (sensitive to methyl group). This conclusion was consistent with the results of HS-SPME-GC-MS, which analyzed that the blank control group detected four alcohols (44.87%), two aldehydes (41.52%), and one ester (6.26%).

**FIGURE 10:**
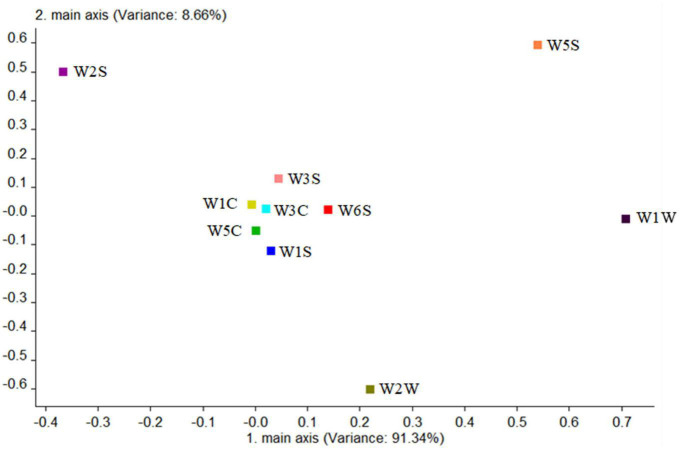
Loadings analysis chart of the odor sensors of the wild-type group.

Second, the contribution rate of the 10 sensors was analyzed in the wild type by Figure 10; it is shown that the W1W sensor (sensitive to many sulfides) had the largest contribution rate in the first principal component, while in the second principal component, the greatest contribution rate was of the W5S sensor (sensitive to nitrogen oxides). The first main component of the wild type was sulfide, and the second principal component was nitrogen oxides mainly. But W1C, W5C, W3C, and W1S sensors had a low contribution rate among them, which can be ignored, indicating that alkane and aromatic substances in this sample were absent or undetectable. It was also consistent with the results of HS-SPME-GC-MS.

**FIGURE 11:**
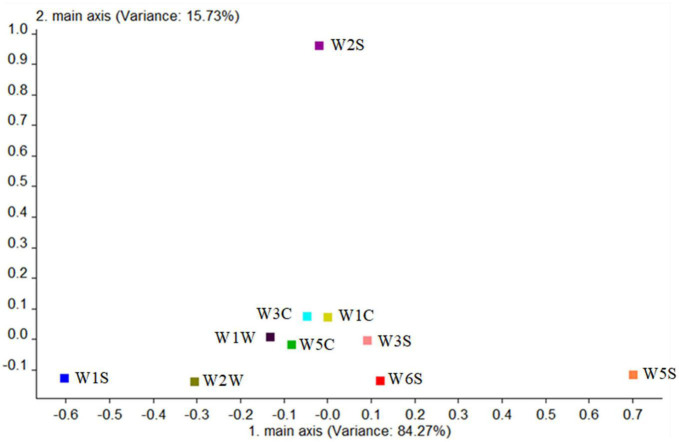
Loadings analysis chart of the odor sensors of the mutagenic-type group.

Finally, the contribution rate of the 10 sensors of the mutagenic type was analyzed in Figure 11, and it is shown that the W5S sensor (sensitive to nitrogen oxides) and W1W sensor (sensitive to many sulfides) had the higher contribution rate in the first principal component, while in the second principal component, the largest contribution rate was the W2S sensor (sensitive to alcohols, aldehydes, and ketones). The first main components of the mutagenic type were sulfide and nitrogen oxides, and the second principal components were alcohols, aldehydes, and ketones.

From the above GC-MS and E-nose analysis, the main aroma-producing substances detected in the samples by the two methods were the same.

Combining the aforementioned loadings analysis with the principal component analysis, it was found that the difference of volatile components between the two strains and the blank control group was mainly on PC1, and the difference between the two strains was mainly on PC2. The following were particularly analyzed: (1) The difference in the contribution rate of PC1 between the two strains and the blank control group was alcohols, sulfides, and nitrogen oxides, indicating the difference between them was the content of these substances, which is consistent with the result of HS-SPME-GC-MS-detected alcohols in the two strains. In addition to alcohols, sulfides and nitrogen oxides were detected because they were precursors of volatile aroma substances fermented by *G. candidum* (Yang et al., 2007). (2) The difference in the contribution rate of PC2 between the two strains was nitrogen oxides, alcohols, aldehydes, and ketones, showing that the difference between the two strains was the content of these substances, which is consistent with those of aldehydes, esters, and phenylethyl alcohol detected in HS-SPME-GC-MS.

## Discussion

There have been many studies on aroma components produced by *G. candidum*, such as the aforementioned. in Daigle et al. (1999), *G. candidum* ATCC 62217 formed fruity aroma compounds on fermented waste bread, including ethyl esters of acetic acid, propionic acid, and butyric acid, which were detected in the wild type or the mutagenic type in this experiment; and Mdaini et al. (2006) found that esters and alcohols were the main fruity flavoring compounds of *G. candidum* isolates, while 2-hexanoic acid ethyl ester reached a high production among them, which was also detected in the two isolates in this experiment. In the research of Wang et al. (2007), it was also found that the main aroma compounds were esters and phenylethyl alcohol of an isolate of *G. candidum*, and the results of the study are consistent with the results of mutagenic-type *G. candidum* in this study, but the composition of its aroma compounds was less than that of mutagenic-type *G. candidum* in this study, and the aroma complexity was also low. In the study of Li et al. (2010), there were 32 aroma substances detected in an isolated of *G. candidum*, including phenylethyl alcohol, but the relative content of it was not high, which was only 3.95%.

Compared with the aforementioned research, the two strains of *G. candidum* in this study have more than 30 kinds of volatile compounds, and the floral and fruity-like aroma complexities of these two strains were higher than those of other strains, indicating that those will be more promising in food additives than other strains of *G. candidum*. In addition, the relative content of phenyl ethyl alcohol of the mutagenic-type *G. candidum* reached 7.63%, which was higher than that of many strains in other studies, indicating that the prospect of the mutagenic type in extracting phenyl ethyl alcohol or directly using living cells was larger than that of others.

In summary, the two isolates of *G. candidum* in this study can produce a pleasant odor after fermentation, and the difference compared with the wild-type of the key odor compound produced by the mutagenic type was phenyl ethyl alcohol, with less research. In many studies, phenyl ethyl alcohol was regarded not only as an additive to flavors and fragrances but also as a biological preservative in food. Therefore, mutagenic-type *G. candidum* had a huge prospect in biological food additives and preservatives. However, phenylethyl alcohol was not detected in the wild-type *G. candidum*. Based on the whole-genome data of the two strains, this study speculates that the key difference between the two strains is the Ehrlich pathway, but its key genes have not been verified in depth, so the follow-up should be combined with RT-qPCR technology to verify the expression of the key genes. Knockout or overexpression methods were used to maximize the fermentation capacity of phenyl ethyl alcohol.

## Conclusion

The two strains of *G. candidum* in this study can produce a pleasant smell after fermentation. The two strains show different aroma notes. The wild type and mutant type are based on fruit aroma and flower aroma, respectively. The reason is that the wild type can produce fruit-like butyl 3-methyl-2-butenoate and isobutyl isovalerate, while the expression of mutant malate dehydrogenase is lower than that of the wild type. This slows down the TCA cycle, and eventually, more L-phenylalanine flows to the Ehrlich pathway to produce rose phenyl ethanol.

## Data availability statement

The datasets presented in this study can be found in online repositories. The names of the repository/repositories and accession number(s) can be found below: NCBI – SAMN27362915 and SAMN27362916.

## Author contributions

JC: conceptualization, investigation, and writing – original draft. QL: conceptualization, methodology, and formal analysis. JW: writing – review and editing. WC: data curation. QKZ: visualization. QPZ: resources. XF: supervision. ZL: validation, project administration, and funding acquisition. All authors contributed to the article and approved the submitted version.

## Conflict of interest

The authors declare that the research was conducted in the absence of any commercial or financial relationships that could be construed as a potential conflict of interest.

## Publisher’s note

All claims expressed in this article are solely those of the authors and do not necessarily represent those of their affiliated organizations, or those of the publisher, the editors and the reviewers. Any product that may be evaluated in this article, or claim that may be made by its manufacturer, is not guaranteed or endorsed by the publisher.
